# FEASIBILITY OF WHEY PROTEIN SUPPLEMENTATION TO IMPROVE OLDER ADULT FUNCTION POST-HOSPITALIZATION

**DOI:** 10.1093/geroni/igae098.3982

**Published:** 2024-12-31

**Authors:** Michelle Yang, Pratik Gongloor, Rachel Deer, Elena Volpi, Shuang Li, Erin Hommel

**Affiliations:** University of Texas Medical Branch, Galveston, Texas, United States; University of Texas Medical Branch, Galveston, Texas, United States; University of Texas Medical Branch, Galveston, Texas, United States; University of Texas Medical Branch, Galveston, Texas, United States; University of Texas Medical Branch, Galveston, Texas, United States; University of Texas Medical Branch, Galveston, Texas, United States

## Abstract

Sarcopenia, the age-related involuntary loss of muscle mass and function, is frequently accelerated following hospitalization. Whey protein supplementation may reduce sarcopenia and improve functional recovery. We performed a pilot randomized-controlled trial to assess feasibility of providing whey protein to older adults during and following an acute hospitalization with the goal to improve function. Subjects ages 65+ were recruited from a single academic hospital’s medical-surgical units. Subjects were randomized into three arms: maltodextrin, whey protein, or collagen supplementation, provided twice daily during hospitalization and for 30-days post-hospitalization. Primary outcomes were recruitment, retention, adherence, and data collection feasibility rates. Secondary outcomes were preliminary efficacy on physical function and body composition. From June 2019 to February 2024, 116 subjects were enrolled, a rate of ~2 per month. Retention was 76.8%. Adherence rates were 58.5% for maltodextrin, 58.6% for whey protein, and 60.6% for collagen. Adherence was significantly higher for subjects completing the study compared to those that withdrew or were re-hospitalized. Data collection feasibility rates were moderate to high with SPPB completed on 100% of participants and DEXA collection on 61% of participants. Preliminarily, there were no statistically significant differences in physical function or body composition between groups. Overall, we demonstrated slow recruitment, low adherence, and moderate retention rates in this study of adults 65+ following an acute hospitalization. A prior trial suggested potential efficacy of whey protein on improving function of frail hospitalized older adults. However, strategies to optimize recruitment, retention, and supplement adherence are necessary before performing further assessment of efficacy.

